# Emerging Roles of MTG16 in Cell-Fate Control of Hematopoietic Stem Cells and Cancer

**DOI:** 10.1155/2017/6301385

**Published:** 2017-11-22

**Authors:** Nickolas Steinauer, Chun Guo, Jinsong Zhang

**Affiliations:** Department of Pharmacology and Physiology, Saint Louis University School of Medicine, St. Louis, MO 63104, USA

## Abstract

MTG16 (myeloid translocation gene on chromosome 16) and its related proteins, MTG8 and MTGR1, define a small family of transcriptional corepressors. These corepressors share highly conserved domain structures yet have distinct biological functions and tissue specificity. In vivo studies have shown that, of the three MTG corepressors, MTG16 is uniquely important for the regulation of hematopoietic stem/progenitor cell (HSPC) proliferation and differentiation. Apart from this physiological function, MTG16 is also involved in carcinomas and leukemias, acting as the genetic target of loss of heterozygosity (LOH) aberrations in breast cancer and recurrent translocations in leukemia. The frequent involvement of MTG16 in these disease etiologies implies an important developmental role for this transcriptional corepressor. Furthermore, mounting evidence suggests that MTG16 indirectly alters the disease course of several leukemias via its regulatory interactions with a variety of pathologic fusion proteins. For example, a recent study has shown that MTG16 can repress not only wild-type E2A-mediated transcription, but also leukemia fusion protein E2A-Pbx1-mediated transcription, suggesting that MTG16 may serve as a potential therapeutic target in acute lymphoblastic leukemia expressing the E2A-Pbx1 fusion protein. Given that leukemia stem cells share similar regulatory pathways with normal HSPCs, studies to further understand how MTG16 regulates cell proliferation and differentiation could lead to novel therapeutic approaches for leukemia treatment.

## 1. Introduction

Since their discovery as recurring participants in leukemia-initiating translocation fusions, the MTG family of transcriptional corepressors has emerged as an important set of regulators regarding cell-fate decisions. As corepressors, these proteins associate with a large variety of known transcriptional complexes to recruit other corepressors and histone modifying enzymes, acting as scaffolds to enhance transcriptional repression and chromatin silencing. The MTG family is comprised of three members: RUNX1T1 (ETO, MTG8, and CBFA2T1), CBFA2T2 (MTGR1), and CBFA2T3 (MTG16, ETO2). This review, however, will adopt the nomenclature most commonly used in the reviewed literature: MTG8 (RUNX1T1), MTGR1 (CBFA2T2), and MTG16 (CBFA2T3). MTG8 is the most extensively researched member of the family; it was first discovered in the context of t(8;21) acute myeloid leukemia (AML), a common cytogenetic distinction of AML that is responsible for 12–15% of total cases [1–3]. t(8;21) fuses the N-terminal, DNA-binding domain of AML1 (RUNX1) to a virtually complete MTG8 fragment, inducing a broad dysregulation of AML1 target genes in preleukemic clones [4–6]. MTGR1 was isolated as a RUNX1-RUNX1T1 (AML1-ETO)-associated protein and immediately identified as a paralog of MTG8 [7]. Finally, cloning and characterization of the t(16;21) breakpoint in rare, treatment-related AML led to the detection of MTG8-homologous sequences on chromosome 16—this gene was named MTG16 (myeloid translocation gene on chromosome 16) [8]. All three MTG family members share four regions of sequence homology that are conserved from the *Drosophila melanogaster* gene *Nervy*. These domains are aptly named “Nervy homology regions” (NHRs), and they mediate the many interactions with DNA-binding transcription factors (TFs) and higher order, corepressor complexes that have come to characterize the MTG family [9, 10]. While the MTG members share these structures, both in sequence and in function, it appears that their individual biological roles are distinct but largely overlapping. Their respective expression in various tissues can be nonspecific (in which all three members are expressed, e.g., in differentiated erythroid cells), or combinatorial (in which only a subset of members is expressed, e.g., hematopoietic stem cells) [11–13]. Many studies have elucidated the binding capabilities of the MTG members, and the consensus seems to be that, generally, the three members are similar, and in most cases redundant, in terms of the proteins with which they can interact. This redundancy is manifested in the fact that knockout of individual MTG members produces relatively mild, yet distinct, phenotypic changes in mice [9]. A likely scenario is that while each of the MTG members may interact with a common set of TFs, corepressors, and histone deacetylases (HDACs), the expression patterns of individual members are different. Another noteworthy feature of the MTG corepressors is their shared ability to form homo- and heterooligomers by virtue of their NHR2 oligomerization domain [7]. Thus, varying combinations of MTG members in these oligomer complexes may regulate varying subsets of target genes or produce varying repressive strengths.

The past two decades of research suggest that these corepressors play a particularly important role in actively differentiating tissues. One of the first studied processes with regard to the MTG family was neural differentiation. When it was first discovered, murine Mtg8 was noted to be most highly expressed in 5-day-old mouse cerebellum—a setting of rapid proliferation and differentiation [14]. A later study found that all MTG members were important in murine neurogenesis. Mtgr1 was expressed in proneural cells, and Mtg16 and Mtg8 transcript levels steadily increased with proneural proliferation and differentiation [15]. Furthermore, adult tissues demonstrating sustained, self-renewal capacity—gut epithelium and the hematopoietic system—are regulated in part by MTG corepressors. The Hiebert group has extensively characterized MTG family knockout mice and their altered phenotypes (Table 1). *Mtg8*^−/−^ mice experience malformation of the midgut, implying a vital role for Mtg8 in gut development [16]. *Mtgr1*^−/−^ mice have deficiencies of the secretory cell lineage in colonic epithelium [17]. Finally, in addition to defects in the hematopoietic system, altered gut immunity and epithelial cell cycling were observed in *Mtg16*^−/−^ mice [18, 19]. MTG functions in transcriptional programming and development have also been extensively studied in the hematopoietic cell compartment of both mice and humans; this has likely been in part spurred on by the recurrent targeting of MTG genes by leukemia-inducing translocations, an implication of their central roles in regulating self-renewal, proliferation, and differentiation by transcriptional means. Within the hematopoietic compartment, MTG16 appears to take a front-seat role, as MTG8 is expressed to a significant degree only in differentiated erythroid cells and several leukemia cell lines (HL60, Kasumi-1, and HEL), while MTGR1 is constitutively expressed at relatively low levels in all hematopoietic cells and is therefore not believed to be serving a major, regulatory role. In contrast, MTG16 is markedly upregulated in hematopoietic stem and progenitor cells [13, 20–22]. A majority of the research body pertaining to MTG corepressors has focused on MTG8 because of its involvement in the t(8;21) translocation, a frequent cause of AML. MTG16, on the other hand, has received little attention by comparison, but its important role in hematopoietic stem cell physiology and pathology is becoming increasingly apparent. The following review will summarize the body of knowledge regarding MTG16 structure, function, physiologic roles, and involvement in human disease.

## 2. MTG16 Gene and Protein Structure

**Figure 1:**
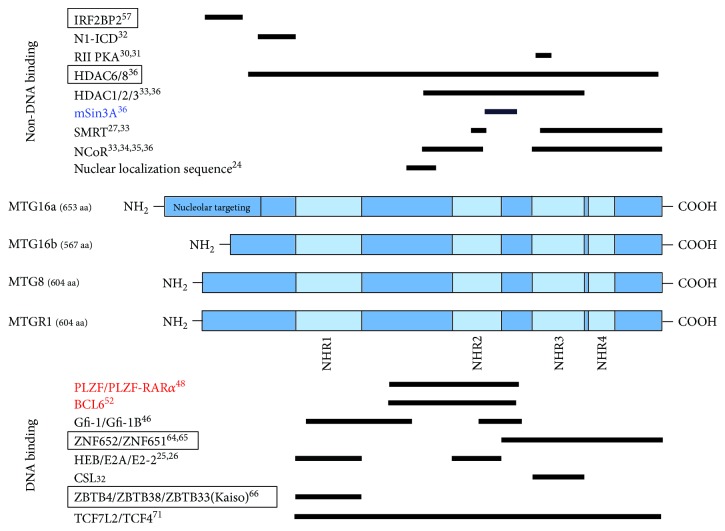
Schematic representations of MTG family proteins and their domain structures. The diagrams are drawn to scale and represent the exact binding domains between MTG proteins and other transcriptional regulators as determined by an extensive literature review. Many of the studies that examine the various MTG corepressor interactions focus on MTG8, and most demonstrate that the given interaction also exists in other MTG family members (MTG16 and MTGR1). In this figure, proteins written in black have been demonstrated to associate with both MTG8 and MTG16 (and MTGR1 in most cases). Proteins in red have been shown to interact with MTG8, but their interaction with MTG16 has never been directly tested. Proteins in blue have been specifically confirmed to interact with MTG8, but not MTG16. Finally, enclosing boxes denote proteins that have been shown to interact with MTG16 but have not been assessed for MTG8 binding.

*MTG16*, officially *CBFA2T3*, is a 15-exon gene that codes for two major transcript variants: MTG16a and MTG16b (Figure 1). These two isoforms differ in their 5′ UTR and the first exon, and they produce separate protein products of differing N-terminal sequences. MTG16a is the longer isoform that codes for a 653-amino acid protein which contains a unique nucleolar localization sequence, destining it for occupation in the nucleolus and nucleoplasm [23]. MTG16b, the shorter of these two isoforms (567 amino acids in length), is constrained to the nucleoplasm [8, 24]. It should be noted that MTG16a is the only MTG member inherently capable of localizing to the nucleolus, although it may recruit other MTG members to this region via physical binding [23].

Like the other members of the MTG family, MTG16 has four, highly conserved regions designated NHR1-NHR4 (Figure 1). NHR1, the most N-terminal of these domains, shares homology with hTAF130 and hTAF105, members of the multimeric, general TF complex, TFIID [14]. Importantly, it is also this region that most significantly interacts with E-proteins, a class of bHLH TFs that recognize E-box consensus sequences (CANNTG), to repress E-protein-dependent activation of target genes [25]. The NHR2 domain contains a hydrophobic heptad repeat (HHR) critical for tetramerization of MTG family members, and it seems that this interaction can occur in the context of homodimeric MTG (e.g., MTG16:MTG16) or heterodimeric MTG (e.g., MTG16:MTG8) complexes [12]. Several lines of evidence suggest that the quaternary structure organized by NHR2 contributes to the repressive function of MTG16. First, the antiparallel dimerization of two NHR2 domains provides a secondary binding interface for E-proteins, which may strengthen MTG association with E-protein complexes or perhaps even direct MTG tetramers to E-protein homodimers (as opposed to E-protein/class II bHLH heterodimers) [26]. Second, NHR2-mediated oligomerization places multiple NHR3/4 domains in close proximity, allowing them to multimerically bind NCoR/SMRT corepressor complexes and effect maximal repression [27]. Finally, deletion of the NHR2 region abolishes the transforming ability of RUNX1-RUNX1T1, hinting that this oligomerization is equally important for the leukemia-initiating mechanism of t(8;21) [28]. Among the MTG family members, NHR3 is the least conserved domain, and relatively, little is known of its function. It does, however, mediate two notable interactions: MTG binding to CSL (CBF1/Su[H]/Lag-1), a nuclear receptor that is converted from a repressor to an activator upon activated Notch signaling, and MTG8/16 binding to the regulatory RII*α* subunit of PKA, making both MTG8 and MTG16 bona fide A kinase anchoring proteins (AKAPs) [29–32]. NHR4 includes two noncanonical zinc finger motifs which mediate interactions with the nuclear corepressors NCoR/SMRT and HDACs rather than DNA binding [33–38]; however, these zinc finger motifs do allow MTG proteins to bind RNA, as a previous study has mapped in vitro RNA interaction to the NHR4- and NHR2-proximal regions of MTG proteins [39]. Several forms of noncoding RNA, including enhancer RNA (eRNAs) and long, noncoding RNA (lncRNAs), have a well-established role in facilitating three-dimensional, cis-regulatory interactions throughout the genome and recruiting chromatin-remodeling complexes [40–42]; thus, the observed interaction between RNA and MTG proteins warrants further investigation. Interestingly, the region between NHR2 and NHR3 mediates Sin3A recruitment by MTG8, but this same interaction is not observed for MTG16 [36]. Another difference between MTG16 and other MTG members is the multitude of HDAC enzymes these corepressors are capable of recruiting. While direct association with HDACs 1-3 is observed with all MTG family members, the ability to interact with HDAC6 and HDAC8 is unique to MTG16 [36].

## 3. Role of MTG16 in Normal Hematopoiesis

In recent years, MTG16 has emerged as a master regulator of normal hematopoiesis in vertebrate animals. Indeed, MTG16 interacts with a growing number of hematopoietic TFs, as well as mediators of Wnt and Notch signaling. Wnt signaling is a key pathway that controls transcriptional programs leading to stem cell self-renewal [43]. Notch signaling is another important pathway that is implicated in several key cell type transitions, including that of hemogenic endothelial cells to hematopoietic stem cells, as well as hematopoietic stem cells to common lymphoid progenitors [44, 45]. We will first review MTG16 regulation of TFs (3.1), followed by a description of MTG16 knockout animal models (3.2) and a discussion of the biological implications of Wnt and Notch regulation by MTG16 (3.3).

### 3.1. MTG16 Is a Corepressor of Diverse Hematopoietic Transcription Factors

**Figure 2:**
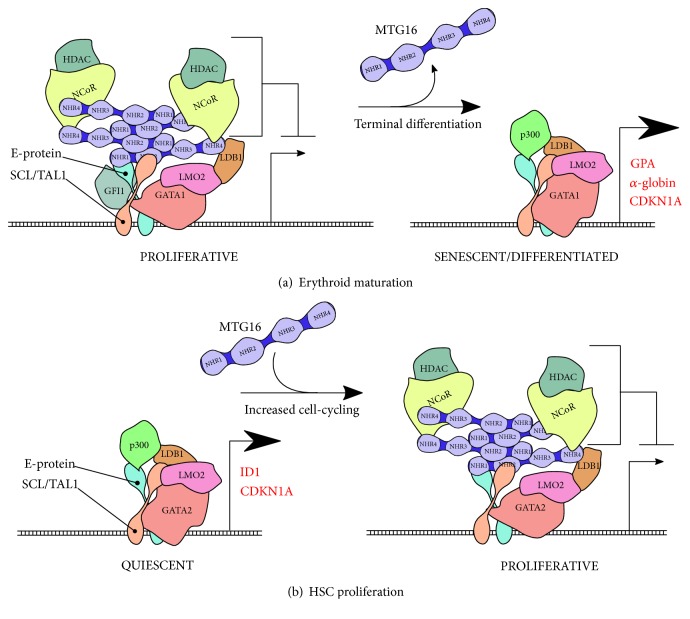
A model of MTG16-mediated repression in the SCL/TAL1:E2A complex. (a) A number of studies concur that MTG16 represses genes poised for rapid activation upon terminal differentiation. MTG16 interacts with the SCL/TAL1:E2A complex in erythroid progenitors and prevents the expression of several erythrocytic genes, including *α-globin*, *GPA*, and *CDKN1A.* The cartoon figure describes the complex dynamics involved in erythroid maturation as described by Schuh et al. [55]. In the erythroid progenitor stage, the complex interacts with MTG16 and Gfi1, but upon differentiation, both MTG16 and Gfi1 dissociate from the complex, allowing for the recruitment of coactivator complexes (p300/CBP) to promote gene transcription. (b). Although not as extensively researched, MTG16 likely plays a similar repressive role in actively dividing HSPCs. The SCL/TAL1 complex activates expression of genes that help to preserve quiescence. Expression of MTG16 can repress these genes and induce a proliferative state in HSPCs. The details of these complex dynamics have not been fully elucidated in HSCs/HSPCs.

MTG16 is a corepressor to two major TF families: zinc finger containing (ZNF, mostly of the C_2_H_2_-type and GATA-type) and basic helix-loop-helix (bHLH) TFs. Many—but not all—of these TFs direct the hierarchical differentiation of hematopoiesis. One early study demonstrated that MTG16 interacts with the Gfi-1 and Gfi-1B zinc finger TFs [46]. Gfi-1 null mice are deficient in long-term and short-term hematopoietic stem cells (LT/ST-HSPCs), which are believed to follow from a loss of quiescence in HSPC pools, given that Gfi-1 also inhibits cell-cycle-inducing genes [47]. The related TF, Gfi-1B, is highly expressed in HSPCs and is depleted in differentiating cells. These two TFs bind to targeted genes and repress their transcription, and it was shown that the presence of MTG8 or MTG16 in these complexes greatly augmented the repression. Before much was known about MTG16, MTG8 was shown to associate with PLZF, a ZNF TF active in HSPCs that can limit cell proliferation and myeloid differentiation [48, 49], as well as BCL6, a sequence-specific, repressive TF that famously coordinates the germinal center reaction of mature B-cells by promoting proliferation, delaying differentiation, and suppressing the *TP53* response to *Ig* V(D)J recombination [50–52]. Although a documented ability of MTG16 to bind these TFs has not been published, these interactions very well may exist given the high structural and sequence homology between MTG members. Direct, stoichiometric binding was also discovered between the MTG8 moiety of the RUNX1-RUNX1T1 fusion protein and E-proteins, a group of class I bHLH TFs [25]. This strong interaction was later confirmed for MTGR1 and MTG16 as well [53]. The basic helix-loop-helix (bHLH) TF family consists of ubiquitously expressed class I bHLHs, otherwise known as E-proteins, and tissue-specific, class II bHLHs which heterodimerize with class I bHLHs to promote binding specificity for tissue-specific gene regulatory regions [54]. MTG16 has been shown to interact with class II bHLH as well, most likely in association with the E-proteins. In erythroblastic and megakaryocytic progenitor cells, MTG16 dynamically regulates the SCL/TAL1:E2A core transcriptional complex, a pentameric structure comprising E2A (class I bHLH), SCL/TAL1 (class II bHLH), LMO2, LDB1, and GATA-1 [55] (Figure 2). In early stages of erythroid and megakaryoid differentiation, MTG16 occupies the SCL/TAL1:E2A complex to repress erythrocytic genes—including *GPA*, *band 4.2*, and *α-globin*—and cell-cycle inhibitor genes such as *CDKN1A* (p21^CIP1/WAF1^). Within these bHLH complexes, the ratio of SCL/TAL1 to MTG16 rapidly spikes, switching the SCL/TAL1 complex to a transcriptional activator, thereby allowing terminal differentiation to proceed [55–57]. Apparently, however, this rapid depletion of MTG16 may not be permanent or universal to all MTG16 target genes, as MTG16, together with LDB1, was found to repress fetal *γ-globin* expression in mature, adult erythroid cells [58]. Thus, it has been proposed that MTG16 regulates the critical developmental window that exists between highly proliferative, progenitor cells and terminally differentiated blood cells [56]. Interestingly, a nearly identical SCL/TAL1:E2A complex with GATA-1 substituted for GATA-2 has been identified in HSPCs and is thought to function in this same manner [59]. SCL/TAL1 has emerged as an especially important facilitator of adult, definitive HSPC formation and a guardian of LT-HSC quiescence; SCL/TAL1 expression closely mirrors that of MTG16 throughout hematopoietic development (high in HSPCs with sustained expression in megakaryocyte-erythroid progenitors) [44, 60]. Furthermore, SCL/TAL1 activates *ID1* and *CDKN1A* expression in the LT-HSC pool, two genes that promote quiescence and can be repressed by MTG16 expression [61].

A recent study examined the role of MTG16 in coordinating the rapid, monocytic burst seen in myocardial infarct patients several days after an ischemic event [62]. The monocytosis was found to be derived from a subpopulation of HSPCs that highly expressed CCR2, a chemokine receptor which directs monocytes from the bone marrow to peripheral sites of infarction [63]. The group analyzed differential gene expression between CCR2^+^ and CCR2^−^ cells and found that the most enriched gene set in CCR2^+^ cells was the set of genes downregulated in *Mtg16^−/−^* LSKs [62]. This finding supports the notion that MTG16 is instrumental in the transition of LT-HSCs to ST-HSCs or MPP (multipotent progenitors) and the proliferative expansion seen with this transition. We therefore favor the model that MTG16 promotes cell cycling in HSPCs (Figure 2). Whether or not MTG16 promotes cell division at the expense of self-renewal is an interesting question worth addressing in future studies.

In the context of breast and colonic epithelium, MTG16 physically interacts with a number of ZNF TFs, including ZNF651, ZNF652, ZBTB4, ZBTB38, and ZBTB33 (Kaiso) [64–66]. Although a detailed description of these interactions is beyond the scope of this review, we have summarized their binding sites within the MTG domains in Figure 1 and will discuss the functional consequences of these interactions in breast and colorectal carcinoma in Section 4.1.

### 3.2. *Mtg16^−/−^* Mouse Model

An invaluable tool for the study of MTG16 has been the creation and subsequent research of *Mtg16*^−/−^ mice (Table 2) [18, 19, 45, 67, 68]. Such mice have no profound anatomical or developmental abnormalities, hinting at the possible functional overlap between MTG family members. *Mtg16*^−/−^ mice do, however, exhibit a dysfunctional hematopoietic system. Lower numbers of B-lymphocytes and megakaryocytic-erythroid progenitors (MEPs) were observed, concurrent with an increased proportion of granulocytic-monocytic progenitors (GMPs) [67]. Upon challenge with phenylhydrazine, a hemolytic agent, these same mice did not exhibit the robust, compensatory hematopoiesis that was seen from the HSPCs of control mice. This was explained by an examination of the LSK (Lin^−^/Sca-1^+^/c-kit^+^, the immunophenotypic designation of murine HSPCs) pool via flow cytometry; expectedly, *Mtg16*^−/−^ mice possessed a smaller population of these cells. Notably, microarray analysis of these LSK populations showed a derepression of target genes for Gfi-1, Bcl-6, and E-proteins [67]. There were also increases in *p27* levels, and exogenous introduction of c-Myc into *Mtg16^−/−^* LSK cells salvaged their repopulating abilities, denoting a role for Mtg16 of inducing proliferation in LSK populations [67]. Unexpectedly, a more recent study showed that LT-HSCs (LSK, Flt3^+^/CD150^+^/CD48^−^) from *Mtg16^−/−^* mice showed overall higher proliferative rates and lower self-renewal capacity, as measured by CFU assays and stem cell transplantation assays [68]. Two possible scenarios may explain this discrepancy. First, it is possible that *Mtg16^−/−^* LSK populations were exhausted due to an earlier proliferation and depletion of LT-HSCs; thus, the cells identified as the LSK population were only Lin^−^/Sca-1^+^/c-kit^+^ in a superficial sense and had lost an intrinsic ability to undergo cell division. Another possibility is that Mtg16 regulates cell division in a context-dependent fashion. This is not at all outside the realm of possibility; indeed, one could speculate on the existence of an LT-HSC-specific orphan factor that may bind to MTG16-containing transcriptional complexes and alter its transcriptional output.

### 3.3. MTG16 as a Suppressor of Wnt and Notch Signaling

Along with these regulatory roles in HSPCs and erythroblastic cells, MTG16 and the other two members of the MTG family show significant crosstalk with the universal cell signaling pathways mediated by Wnt and Notch. These pathways allow integration of extracellular information with transcriptional programming and are key mediators of self-renewal and multicellular development. Wnt and Notch signals have been implicated in the instruction of stem cell self-renewal, and the fact that MTG16 interacts with both of these pathways, and a large number of hematopoietic master TFs, implies a role for MTG16 as an orchestrator of self-renewal and differentiation in HSPCs [69].

**Figure 3:**
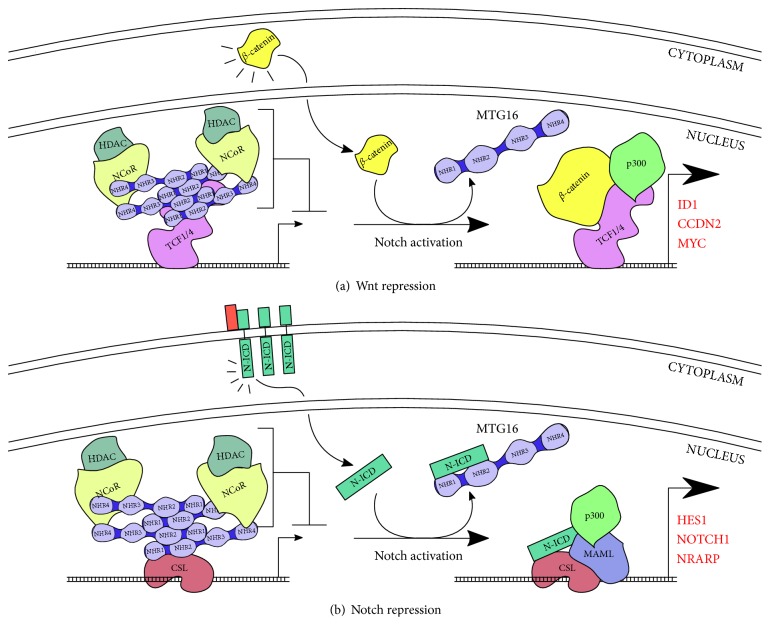
MTG16, and other MTG family members, may play a role in the constitutive repression of both Wnt and Notch signaling pathways. (a) Cell surface activation of Frizzled-family receptors attenuates the constitutive phosphorylation and degradation of *β*-catenin in the cytoplasm. An increase in *β*-catenin concentration and translocation to the nucleus convert TCF-family TFs to activators of transcription at Wnt target genes. MTG family members were shown to bind Tcf4 in vitro, and the association was abrogated in the presence of *β*-catenin, lending credence to the idea that MTG family members represent one of the many corepressors employed by TCF TFs in basal, inactivated states. The specific contribution of MTG16 to this pathway is controversial, and the most recent research suggests that MTGR1 carries most of the Wnt-modulatory roles in the MTG family. (b) MTG16 also interacts strongly with the intracellular domains of all four mammalian Notch receptors (N-ICD). Similar to Wnt signaling, Notch activation induces proteolytic cleavage of the intracellular portion of the receptor and translocation to the nucleus. It then converts the CSL repressor into an activator. MTG16 interacts with CSL but is displaced by N-ICD. N-ICD also directly binds MTG16, likely inducing a conformational change that inhibits MTG16:CSL interaction. Engel et al. [32] demonstrated a role of endogenous MTG16 in Notch-directed T-cell differentiation from HSPCs.

When the canonical Wnt signaling cascade is activated, cytoplasmic *β*-catenin is protected from phosphorylation by the Axin complex (Axin/APC/CK1/GSK3). While the steady-state phosphorylation of *β*-catenin triggers its proteasomal degradation, nonphosphorylated *β*-catenin accumulates and eventually translocates to the nucleus. Here, it associates with TEL/TCF TFs, which are primed and waiting at target gene promoters, to activate transcription [70]. In the absence of *β*-catenin, TEL/TCF factors strongly associate with corepressors to prevent Wnt-activated transcription (Figure 3(a)). Murine Mtgr1 associates with Tcf4 and Tcf1 in vitro and can repress the expression of a Tcf4-dependent reporter gene. Immunoprecipitation experiments further show that Tcf4 pulldown is preserved with Mtg8 and Mtg16 constructs, although to a considerably weaker degree [71]. While these results suggest that Mtg16 can physically associate with transcription factors of the Wnt pathway, it was not conclusively demonstrated that Mtg16 plays a significant role in endogenous Wnt signaling. Indeed, in murine LSK HSPCs, Mtg16 knockout results in upregulation of only two Wnt target genes (*Ccdn2* and *Id1*) [67]. A recent study in the *Apc^1638/+^* mouse model, which replicates familial adenomatous polyposis (FAP) and features intestinal epithelium prone to Wnt dysregulation and colorectal polyps/carcinoma, showed that deletion of Mtgr1, but not Mtg16, increased tumor multiplicity tenfold. ChIP-Seq datasets for Mtgr1 and Mtg16 in mouse erythroid leukemia cells revealed widespread binding of Mtgr1 (1388 significant recovered peaks) and a much more selective binding of Mtg16 (353 peaks, of which 325 were shared with Mtgr1) [72]. Gene ontology analysis of these Mtgr1-bound genes revealed a significant enrichment of Wnt and Notch target genes, which was not observed in the set of Mtg16-regulated genes [72, 73].

Notch signaling is another key developmental pathway associated with MTG16 (Figure 3(b)). Particularly, it regulates the emergence of the HSPC population from hemogenic endothelial cells of the aorta-gonad-mesonephros (AGM) during embryonic, definitive hematopoiesis [74, 75], multiple stages of lymphocytic differentiation [76], and LT-HSC self-renewal and quiescence in the bone marrow niche [77]. Mammals possess four Notch receptors which are cell membrane bound but become proteolytically cleaved upon receptor-ligand binding [78]. These cleaved intracellular fragments, known as Notch-intracellular domains (N-ICD), translocate to the nucleus, bind to the CSL (CBF1/Su[H]/Lag-1) transcriptional complex, and derepress its transcription in a manner similar to Wnt transcriptional activation [78]. A connection between Notch signaling and Mtg16 was found when Mtg16*^−/−^* mouse LSK cells showed an upregulation of Notch-target genes, specifically *Hes1*, *Notch1*, and *Nrarp*. This prompted the authors to test for physical interactions between Mtg16 and Notch proteins; remarkably, they discovered interactions between Mtg16 and CSL and Mtg16 and N1-ICD (the intracellular domain of the Notch1 receptor). The addition of N1-ICD to the system abrogated any detectable interaction between Mtg16 and CSL, proposing a mechanism by which N1-ICD sequesters Mtg16 to prevent its binding to and repression of the CSL complex [32]. Notch receptors and their ligands of the Delta or Jagged/Serrate families couple juxtacrine signals to transcriptional responses. This process is important for definitive hematopoiesis, the phenomenon by which clusters of endothelial cells within the embryonic, dorsal aorta transition to hematopoietic stem cells [78]. Importantly, these HSPCs are distinctively capable of maintaining long-term hematopoiesis, a feature that sets them apart from the transient, hematopoietic cells arising from primitive hematopoiesis [79]. Notch signaling input is crucial in the specification of hemogenic endothelium from arterial endothelium, and this specification appears to be mediated by *Gata2*, *Hes1*, *Hes5*, and *Hey2*; all of which are Notch target genes [78]. Counterintuitively, a transient suppression of Notch signaling is required after specification for HSPC emergence—this may be executed by MTG16, given its ability to repress Notch transcription and the observation that MTG16 localization changes from cytoplasmic and nuclear to solely nuclear around this time period in zebrafish definitive hematopoiesis (a highly conserved process that is mostly analogous to human definitive hematopoiesis) [80, 81]. Studies of *Xenopus* development have uncovered an additional, second arm of HSPC emergence directed through Vegfa regulation. Vegfa is dually implicated in directing hemangioblasts towards arterial, endothelial differentiation and subsequent HSPC emergence. MTG16 was found to activate Vegfa expression in somites, demonstrating an additional non-cell-autonomic effect of this important corepressor [82]. The sheer complexity and density of these hemogenic transcriptional networks limit a precise understanding of MTG16 function in HSPC emergence, but the overall effect of MTG16 knockout in zebrafish is a severe reduction in definitive hematopoiesis [80]. Provided MTG16 does repress Wnt and Notch gene transcription in the absence of pathway activation, MTG16 overexpression does not necessarily have an inverse, negative effect on Wnt- and Notch-mediated self-renewal, and MTG16 expression may only ensure Wnt and Notch activation is specific, potent, and transient.

## 4. MTG16 in Carcinoma and Leukemia

### 4.1. MTG16 Is a Putative Tumor Suppressor in Breast and Colorectal Carcinomas

Given the described intersections of MTG16 with hematopoietic transcriptional programs, as well as its regulation of the Wnt and Notch signaling pathways, it is not surprising that this corepressor has also been associated, in a context-dependent manner, with cancers of diverse origins such as the hematopoietic system, colon, and breast epithelium. Early on, MTG16 was reported to be a tumor suppressor in ductal carcinoma of the breast [83]. In fact, loss of heterozygosity (LOH) at the *CBFA2T3* locus (16q24.3) is observed in an estimated 36–67% of ductal carcinomas, and ectopic overexpression of MTG16 in breast cancer cell lines inhibits their colony formation [83]. MTG16 is also downregulated in colorectal carcinoma (CRC), where it plays an important role in Kaiso-directed repression of the colon cancer promoting gene *MMP-7* [66, 84]. MTG16 was recently found to downregulate genes encoding glycolytic enzymes while promoting oxygen-dependent respiration. It does so by enhancing the degradation of HIF1*α*, an oxygen-sensing TF that diverts cells to a glycolytic state in situations of hypoxia [85, 86]. Cancer cells, and other cell types with high proliferative indices, preferentially utilize glycolytic metabolic pathways to derive ATP [87]. Thus, regulating the expression of glycolytic enzymes could be one of the mechanisms by which MTG16 functions as a tumor suppressor in these carcinomas. Interestingly, however, the Warburg effect seems to be partially reversed in both normal and malignant hematopoietic tissues. The bone marrow niche is subject to low pO_2_ tension, and quiescent HSPCs subsist almost entirely by anaerobic glycolysis [88]. The few proliferative HSPCs in this environment dramatically increase their mitochondrial respiration and oxygen consumption upon cell cycling. Several studies suggest leukemia cells prefer this proliferative, oxidative state and have shown that high OXPHOS activity predicts chemotherapeutic resistance and poorer prognoses [89, 90].

### 4.2. MTG16 in Hematopoietic Neoplasms

MTG16 is also targeted by several translocations that cause hematopoietic neoplasms, and this is the gene's namesake (myeloid translocation gene 16). For example, t(16;21) fuses the N-terminal region of RUNX1 (AML1) to the C-terminal region of MTG16 and is seen in a rare form of therapy-induced AML. Additionally, in 30% of pediatric non-Down syndrome acute megakaryoblastic leukemia (non-DS AMKL), inv(16)(p13.3q24.3) fuses MTG16 to the DNA-binding domain of GLIS2 (ETO2-GLIS2) [91]. This fusion TF upregulates BMP2 and genes related to the Hedgehog signaling pathway, leading to enhanced self-renewal. Murine HSPCs that were transduced with ETO2 (MTG16), GLIS2, or ETO2-GLIS2 showed an increase in KIT^+^ HSPCs, CD41^+^CD42^+^ mature megakaryocytes, and CD41^+^CD42^−^ immature megakaryocytes, respectively [92]. This suggests that the MTG16 moiety of the ETO2-GLIS2 fusion is responsible for preserving a HSPC-like phenotype and delaying megakaryocytic maturation. This same study found that expressing a dominant-negative NHR2 peptide could block ETO2-GLIS2 oligomerization and promote differentiation of AMKL blasts [92]. Finally, another translocation, t(14;16), was found in two cases of pediatric B-cell lymphoma [93]. This places MTG16 proximal to the *IGH* promoter and results in supraphysiologic levels of MTG16 expression in these clones. Marked MTG16 upregulation is also seen in patient samples expressing the Ig-IRF4 fusion; this implies an oncogenic role for MTG16 despite its known tumor suppressor characteristic in epithelial tissues [93]. Interestingly, a tumor suppressor function of MTG16 may also be involved in a form of acute lymphoblastic leukemia (ALL). A recent work showed that among the three E-protein members (E2A, HEB, and E2–2), E2A has a reduced binding affinity (relative to its family member HEB) for MTG16 due to E2A-specific changes of amino acids in the MTG16-binding pocket [53]. E2A, including its MTG16-interacting region, can be pathologically fused to other TFs (PBX1 and HLF1). The E2A fusion proteins are thought to drive leukemia development by activating their target genes, and this ability is facilitated by the reduced binding affinity between E2A and MTG16. Replacing the MTG16-binding region of E2A with that of HEB completely abolished the ability of E2A-PBX1 to activate oncogenic target genes and transform cells. These studies suggest that approaches to enhance the binding affinity between MTG16 and E2A could potentially cure the leukemias caused by E2A fusion proteins [53]. For the fusion proteins directly involving MTG16 or other MTG members (RUNX1-RUNX1T1, RUNX1-CBFA2T3, and ETO2-GLIS2), the NHR2 domain seems to be vital for leukemia induction. Thus, small-molecule inhibitors of NHR2-dependent oligomerization may promote differentiation or apoptosis of leukemic blasts, similar to the experience with PML-RARA and all trans retinoic acid (ATRA) therapy [94]. MTG-targeting fusion proteins are of the most common translocations in leukemia, and targeted therapy against these fusions would be useful for a large AML patient population. Finally, small-molecule inhibitors of endogenous MTG16 function could potentially be effective in the general treatment of AML, as most research seems to converge upon the idea that MTG16 promotes proliferation and stalls differentiation in a continuum of hematopoietic cell types. The observation that *Mtg16^−/−^* mice are viable and have a normal output of *steady-state* hematopoiesis suggests this transcriptional corepressor could be temporarily targeted (e.g., by an adjuvant with traditional chemotherapy) with minimal toxicity towards physiologic hematopoiesis.

## 5. Conclusions

The MTG family has been conserved throughout evolution, dating back to invertebrate origins—this most certainly implies a central role for these transcriptional corepressors in cell development. As a regulator of bHLH, PLZF, BCL6, Gfi-1, and GATA TFs, MTG16 is an important coordinator of both primitive (HSPCs) and maturing (megakaryocyte-erythroid progenitors) hematopoietic phenotypes. MTG16's secondary integration with Wnt and Notch pathways hints at some connection with self-renewal and stem cell function. Given that MTG16 is the most highly expressed MTG corepressor in HSPCs [13], it may modulate the self-renewal of HSPCs exiting the LT-HSC reserve, coupling this process to the proliferative increase seen in MPPs. MTG16 may also function as a gatekeeper and a check-point protein to ensure that genes are repressed prior to their needed activation, thus preventing premature differentiation of stem cells. One glaring question remains, however: How do we account for the polar differences of MTG16 function seen in different cancers and tissues? MTG16 is likely to play context-dependent roles in different cancers and leukemias or in different stages of the cancer/leukemia development. Any effective therapies targeting MTG16 must be preceded by sufficient understanding of the mechanisms that MTG16 plays in that particular cancer type. With regard to the actual strategy to be used to target MTG16, given that MTG16 does not directly bind to DNA, the high-level, biological function of MTG16 should be fully dependent on the DNA-binding factors expressed in a particular cell type. Therefore, blocking the interactions of MTG16 (or MTG16 fusion proteins) with the wild-type transcription factors, such as E-proteins, or strengthening the interactions of wild-type MTG16 with E-protein-containing leukemia fusion proteins, such as E2A-PBX1, may be considered for the treatment of the related diseases. Small molecules or peptidomimetics are promising directions, but their rationalized development may need 3D structures of MTG16 with and without its binding partners. Solving these structures should be an important future direction regarding MTG16. Given the emerging technology of CRIPSR, it may also be possible to alter the function of MTG16 by disrupting or enhancing its interactions with E-proteins or E2A fusion proteins, based on protein-protein interaction and structural information. Finally, because MTG16 binding is dependent on many other nuclear proteins, its sum effect in a cell is likely a function of many variables. Indeed, the integration of many key biological pathways with MTG proteins supports the idea that MTG16 and other MTG members are important “hub” genes in complex transcription networks. A holistic appreciation of these networks will hopefully lead to a comprehensive understanding of the MTG corepressors in hematopoiesis and cell development at large and also foster the development of novel therapeutic targets for the treatment of leukemia.

## Figures and Tables

**Table 1:** The MTG family gene nomenclature.

Name	Official name	Alternative names	Reference
MTG16	*CBFA2T3*	ETO2, MTGR2, RUNX1T3, ZMYND4	[12]
MTG8	*RUNX1T1*	CDR, ETO, MTG8, AML1T1, ZMYND2, CBFA2T1	[6]
MTGR1	*CBFA2T2*	EHT, p85, MTGR1, ZMYND3	[7]
RUNX1-RUNX1T1	*RUNX1-RUNX1T1*	AML1-ETO, RUNX1-MTG8	[4]

**Table 2:** Mtg family knockout mice have varying but related phenotypes.

Gene	Genetic background	Targeted exon	Mouse knockout phenotype	References
*Mtg16*	SvEv129 X C57BL/6	8	(i) Mild anemia and reticulocytosis (ii) Extramedullary hematopoiesis during neonatal growth (iii) Reduced numbers of B-cells, megakaryocytes, and erythroid progenitors (iv) Increased numbers of granulocyte/macrophage progenitors (CFU-G) (v) Highly sensitive to phenylhydrazine-induced hemolysis (vi) Unable to undergo compensatory, stress erythropoiesis (vii) Reduced absolute number of LSK HSPC population, relative increase in CMPs and GMPs, relative reduction in MEPs (viii) Loss of quiescence in LSK CD150^+^ CD48^−^ (LT-HSPCs) (ix) Cell intrinsic decrease in stem cell self-renewal (x) Increased proliferation and apoptosis of colonic epithelial cells (xi) Exaggerated immune response following chemically induced colitis (xii) Increased regenerative capacity in intestinal crypt stem cells following ionizing radiation-induced colitis	[18, 19, 45, 67, 68]

*Mtg8*	C57BL/6	2	(i) 25% of homozygous knockouts show deletion of the midgut (ii) Blunted colonic villi and dilated lumen (iii) 30–50% smaller than littermate controls (iv) Male sterility	[16]

*Mtgr1*	SvEv129 X C57BL/6	7	(i) 15–20% smaller than littermate controls (ii) 50% of embryos die E18.5–P21 (iii) Progressive loss of the pansecretory lineage of colonic epithelium (iv) Mild inflammatory infiltrate in gut	[17]
